# Supplementary material for: The Effect of Fibulin-5 on Hydrocephalus After Subarachnoid Hemorrhage in Mice
Source: Int J Mol Sci. 2025 Aug 26;26(17):8259. doi: 10.3390/ijms26178259 (PMC12428664; doi:10.3390/ijms26178259)
Supplement: Supplementary file 1 [file ijms-26-08259-s001.zip › ijms-3805608-supplementary.pdf]

Supplementary Material

Supplementary Tables and Figures

International Journal of Molecular Sciences

The Effect of Fibulin-5 on Hydrocephalus After Subarachnoid Hemorrhage in Mice

Yume Suzuki, Mai Nampei, Fumihiro Kawakita, Hiroki Oinaka, Hideki Nakajima,

and Hidenori Suzuki

Address correspondence to: Hidenori Suzuki, M.D., Ph.D.

Department of Neurosurgery, Mie University Graduate School of Medicine, 2-174 Edobashi, Tsu, Mie 514-8507, Japan

E-mail: suzuki02@med.mie-u.ac.jp

ORCID: 0000-0002-8555-5448

Supplementary Table S1 The number of animals and mortality in experiment 1

Modeling	Treatment	Mortality	Mild SAH	Total mortality	p value
Sham	PBS	0/12 (0.0%)	0	0/12 (0.0%)	
SAH	PBS	1/16 (6.3%)	3	6/47 (12.8%)	0.485
	Short rFBLN5 0.01μg	2/8 (25.0%)	0		
	Short rFBLN5 0.1μg	0/7 (0.0%)	1		
	Long rFBLN5 0.01μg	2/9 (22.2%)	1		
	Long rFBLN5 0.1μg	1/7 (14.3%)	0		

Data, the number of dead mice / total number of mice (mortality). Mild subarachnoid hemorrhage (SAH) is defined as SAH grading score ≤7. PBS, phosphate-buffered saline; rFBLN5, recombinant fibulin-5. Fisher’s exact test is used to compare the mortality of each SAH group

Supplementary Table S2 The number of animals and mortality in experiment 2

Modeling	Treatment	Mortality	Mild SAH	Total mortality	p value
Sham	PBS	0/16 (0.0%)	0	0/16 (0.0%)	
	PBS	19/39 (48.7%)	4		
	Short rFBLN5 0.01μg	3/15 (20.0%)	4		
SAH	Short rFBLN5 0.1μg	4/14 (28.6%)	1	27/84 (32.1%)	0.028
	Long rFBLN5 0.01μg	0/7 (0.0%)	1		
	Long rFBLN5 0.1μg	1/9 (11.1%)	1		

Data, the number of dead mice / total number of mice (mortality). Mild subarachnoid hemorrhage (SAH) is defined as SAH grading score ≤4. PBS, phosphate-buffered saline; rFBLN5, recombinant fibulin-5. Fisher’s exact test is used to compare the mortality of each SAH group

Supplementary Table S3 The number of animals and mortality in experiment 3

Modeling	Treatment	Mortality	Mild SAH	Total mortality	p value
Sham	PBS	0/6 (0.0%)	0	0/6 (0.0%)	
SAH	PBS	2/8 (25.0%)	0	5/23 (21.7%)	0.370
	Long rFBLN5 0.01μg	0/6 (0.0%)	0		
	Long rFBLN5 0.1μg	3/9 (33.3%)	0		

Data, the number of dead mice / total number of mice (mortality). Mild subarachnoid hemorrhage (SAH) is defined as SAH grading score ≤4. PBS, phosphate-buffered saline; rFBLN5, recombinant fibulin-5. Fisher's exact test is used to compare the mortality of each SAH group

Supplementary Table S4 The number of animals and mortality in experiment 4

Modeling	Treatment	Mortality	Mild SAH	Total mortality	p value
Sham	PBS	0/6 (0.0%)	0	0/6 (0.0%)	
	PBS	5/15 (33.3%)	3		
SAH	Long rFBLN5 0.01μg	0/7 (0.0%)	1	6/31 (19.4%)	0.217
	Long rFBLN5 0.1μg	1/9 (11.1%)	1		

Data, the number of dead mice / total number of mice (mortality). Mild subarachnoid hemorrhage (SAH) is defined as SAH grading score ≤4. PBS, phosphate-buffered saline; rFBLN5, recombinant fibulin-5. Fisher's exact test is used to compare the mortality of each SAH group

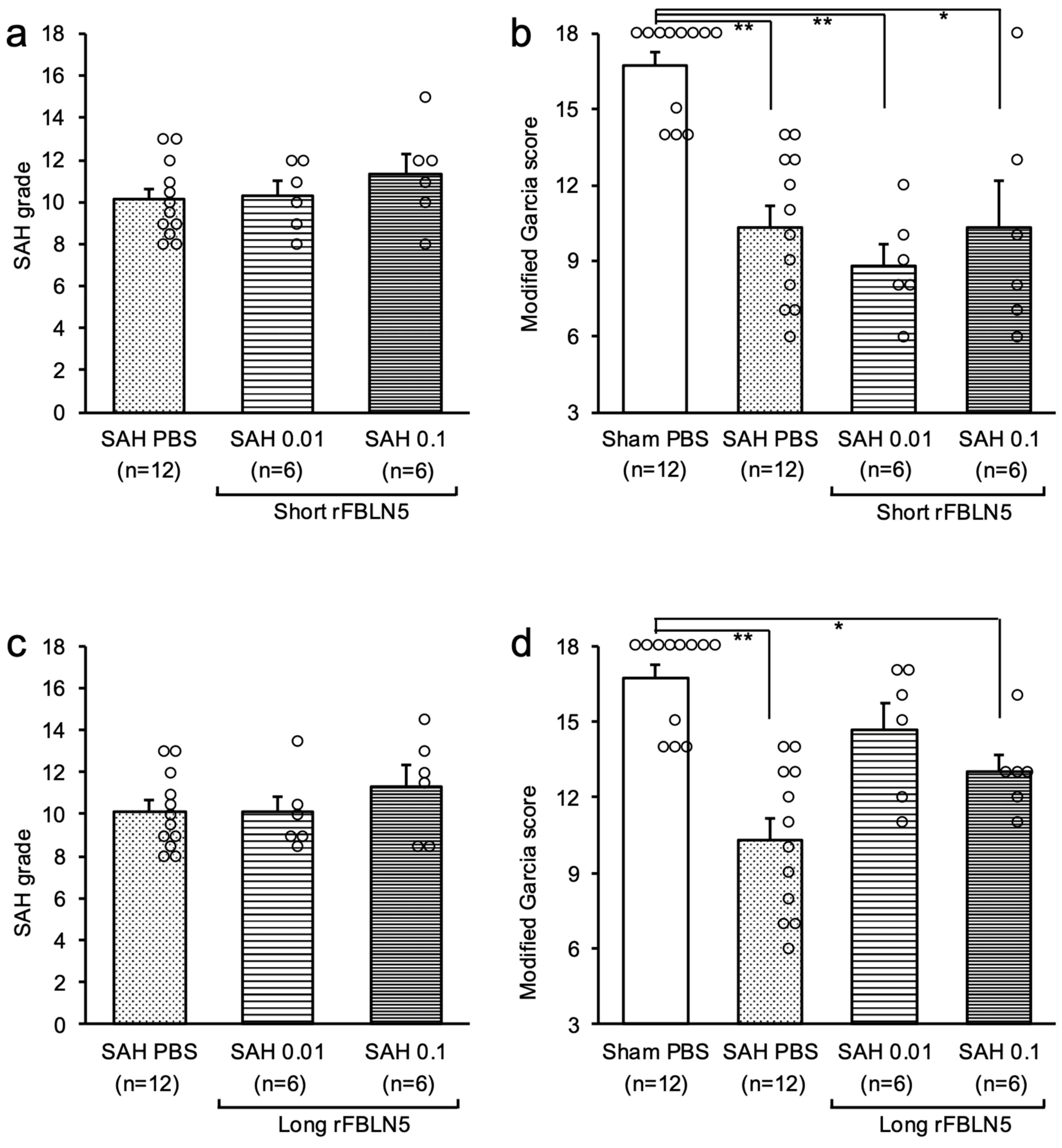

Supplementary Fig. S1 The effects of 2 dosages (0.01 or 0.1μg) of recombinant fibulin-5 (rFBLN5) administration on subarachnoid hemorrhage (SAH) grade (**a**, **c**) and neurological score (**b**, **d**) at 24 hours after SAH. To reduce the number of mice sacrificed, sham phosphate-buffered saline (PBS) mice and SAH PBS mice are duplicated in both analyses. *p<0.05 vs Sham PBS group, and **p<0.01 vs Sham PBS group; Kruskal-Wallis test

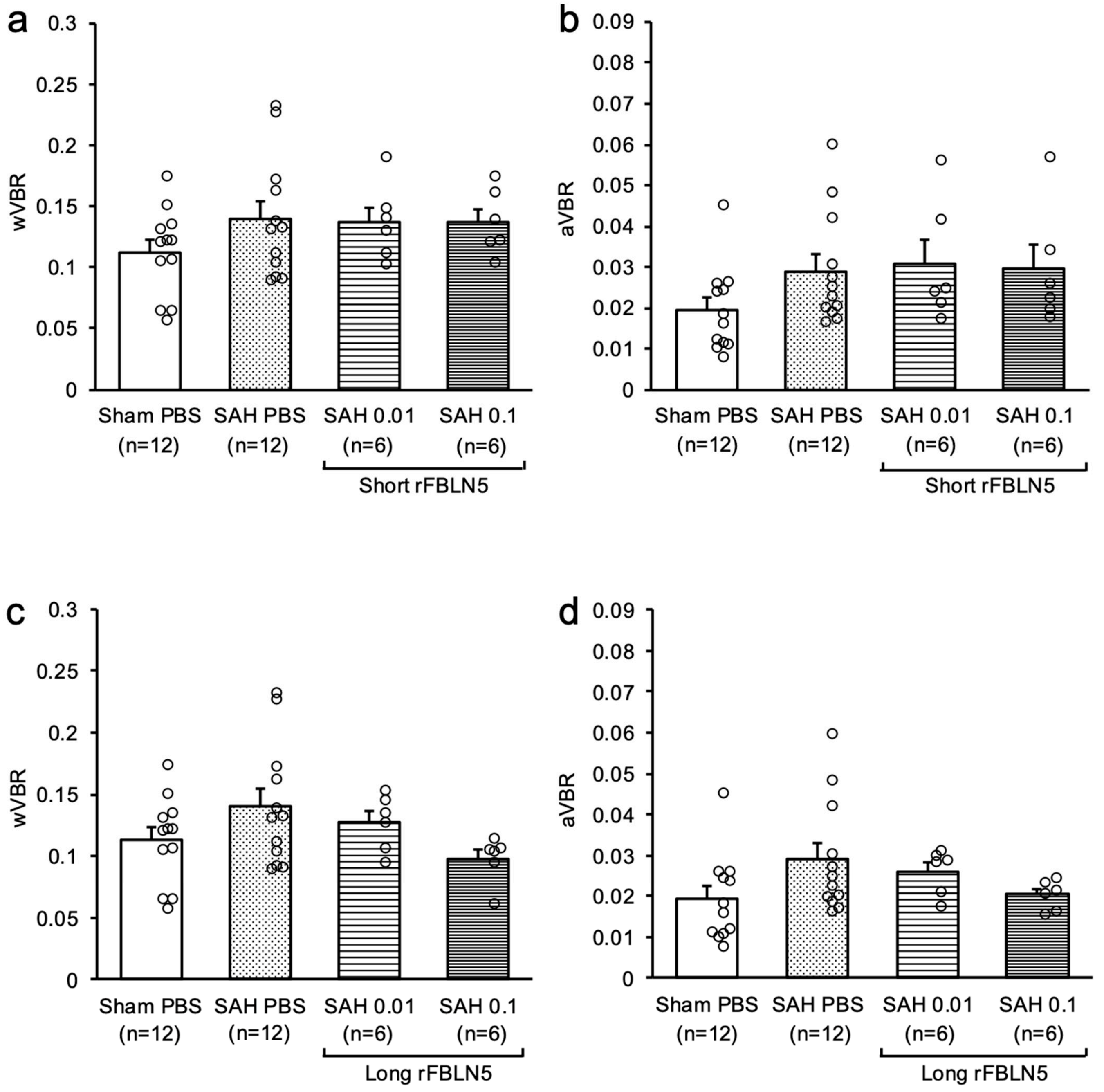

Supplementary Fig. S2 The effects of two dosages (0.01 or 0.1μg) of administered short or long recombinant fibulin-5 (rFBLN5) on ventricle-to-brain ratio at 24 hours after subarachnoid hemorrhage (SAH). There are no significant differences among the groups (**a**, one-way ANOVA; **b**–**d**, Kruskal-Wallis test). aVBR, area of ventricle-to-brain ratio; PBS, phosphate-buffered saline; wVBR, width of ventricle-to-brain ratio

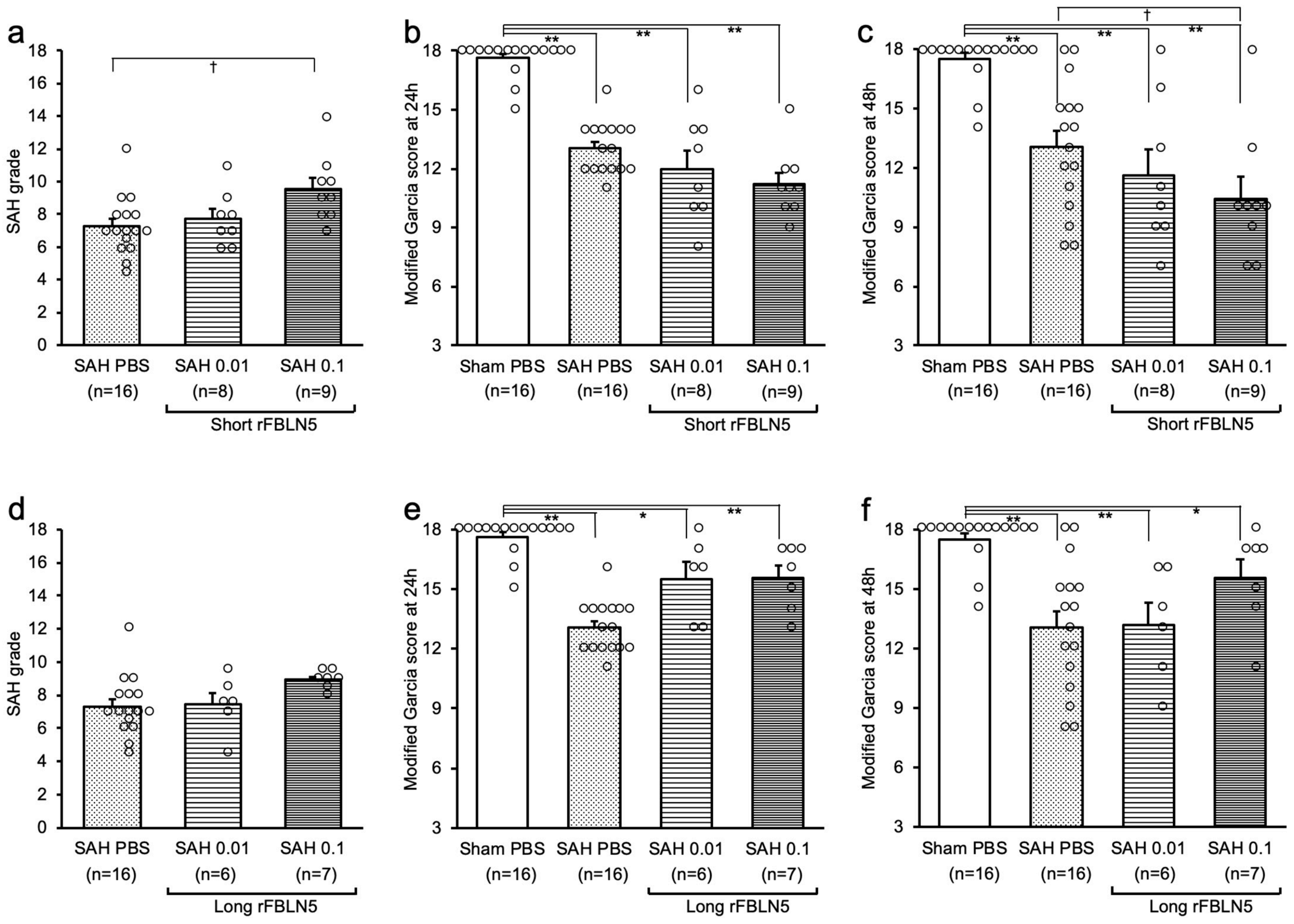

Supplementary Fig. S3 The effects of 2 dosages (0.01 or 0.1μg) of recombinant fibulin-5 (rFBLN5) administration on subarachnoid hemorrhage (SAH) grade (**a**, **d**) and neurological score at 24 hours (**b**, **e**) and at 48 hours (**c**, **f**) after SAH. To reduce the number of mice sacrificed, sham phosphate-buffered saline (PBS) mice and SAH PBS mice are duplicated in both analyses. *p<0.05 vs Sham PBS group, **p<0.01 vs Sham PBS group, and †p<0.05 vs SAH PBS group; Kruskal-Wallis test

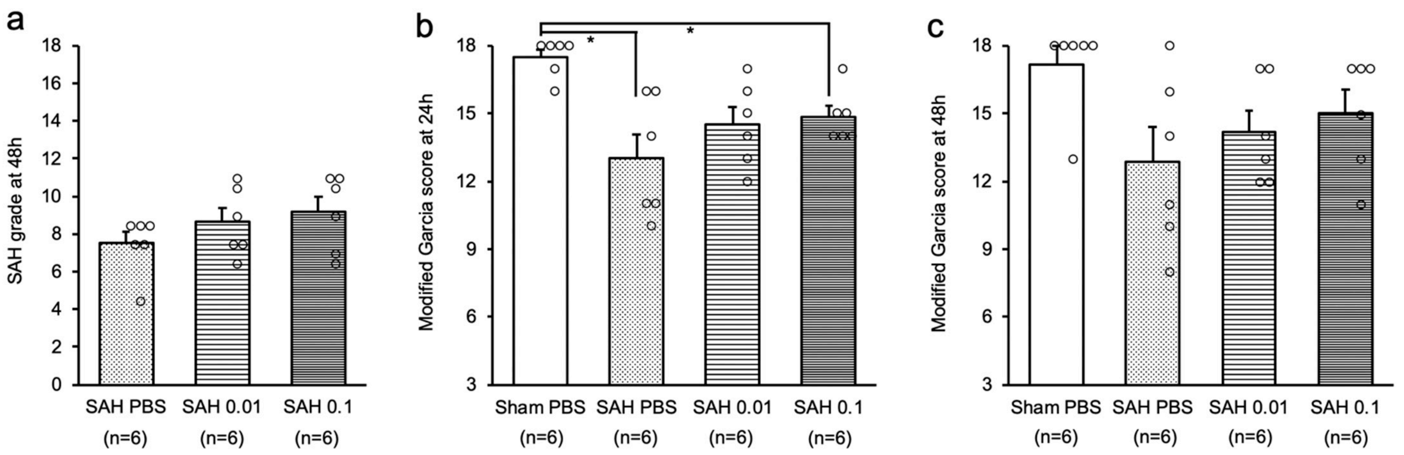

Supplementary Fig. S4 The effects of 2 dosages (0.01 or 0.1μg) of long recombinant fibulin-5 administration on subarachnoid hemorrhage (SAH) grade (**a**) and neurological score at 24 hours (**b**) and at 48 hours (**c**) after SAH. *$p<0.05$ vs Sham phosphate-buffered saline (PBS) group; Kruskal-Wallis test
